# Multimodal Imaging of Dynamic Functional Connectivity

**DOI:** 10.3389/fneur.2015.00010

**Published:** 2015-02-16

**Authors:** Enzo Tagliazucchi, Helmut Laufs

**Affiliations:** ^1^Institute for Medical Psychology, Christian Albrechts University, Kiel, Germany; ^2^Department of Neurology and Brain Imaging Center, Goethe University Frankfurt, Frankfurt, Germany; ^3^Department of Neurology, University Hospital Schleswig Holstein, Kiel, Germany

**Keywords:** dynamic connectivity, functional connectivity, resting-state, EEG, fMRI, EEG–fMRI, wakefulness, sleep

## Abstract

The study of large-scale functional interactions in the human brain with functional magnetic resonance imaging (fMRI) extends almost to the first applications of this technology. Due to historical reasons and preconceptions about the limitations of this brain imaging method, most studies have focused on assessing connectivity over extended periods of time. It is now clear that fMRI can resolve the temporal dynamics of functional connectivity, like other faster imaging techniques such as electroencephalography and magnetoencephalography (albeit on a different temporal scale). However, the indirect nature of fMRI measurements can hinder the interpretability of the results. After briefly summarizing recent advances in the field, we discuss how the simultaneous combination of fMRI with electrophysiological activity measurements can contribute to a better understanding of dynamic functional connectivity in humans both during rest and task, wakefulness, and other brain states.

## Introduction

Functional connectivity is a broad term used to denote statistical co-variation between activity time series in different brain regions (1). It should not come as a surprise that functional connectivity is not static but under constant change. If functional connectivity is considered a proxy for how tightly brain regions interact, then static values represent a fixed scheme for the transmission of information between cortical areas. This view is incompatible with the dynamic nature of the challenges posed by our environment. Even during rest and sleep static connectivity cannot be expected, since spontaneous activity re-capitulates the patterns observed during task performance and sensory stimulation (2).

KEY CONCEPT 1. Functional connectivity This concept refers to signals of neural origin that co-vary over time, for instance, two regions showing similar temporal patterns of spontaneous activity fluctuations. In fMRI signals, functional connectivity is commonly measured by linear correlation. Functional connectivity does not imply a causal mechanism between the signals.

However, until recently functional magnetic resonance imaging (fMRI) studies focused on functional connectivity as computed from signals extracted over extended periods of time. In the case of resting-state analyses this usually corresponds to the whole duration of the scanning session (commonly 5–10 min). This is in contrast with other neuroimaging modalities such as electroencephalography (EEG) and magnetoencephalography (MEG), for which long-dated precedents on how to measure and interpret dynamic changes in functional connectivity exist [see for instance (3)]. The relatively late blooming of assessing the temporal dynamics of connectivity using fMRI is likely based on preconceptions about the limitations of the technique. Compared to MEG and EEG, fMRI has a relatively slow temporal sampling rate. With one measurement every 2 s (a typical value) a 10 min session would yield 300 data points. Van Dijk et al. showed that connectivity estimates become unreliable when computed over windows shorter than approximately 4 min (4). In this context, “reliability” is relative to the static connectivity estimates computed using whole time series. Additionally, electrophysiological activity is represented in blood oxygen level dependent (BOLD) signals as convolved with the hemodynamic response function, which acts as a low-pass filter and lags the BOLD response (5). At the core of using long time series for computing connectivity estimates are the following assumptions: (i) short temporal windows result in noisy estimates of connectivity, (ii) very short temporal windows might fail to disambiguate different neuroelectrical contributions due to their convolution with the hemodynamic response function, and (iii) longer time series are able to capture a temporal average of the short-term dynamics, i.e., a stationary state.

KEY CONCEPT 2. fMRI and BOLD Functional Magnetic Resonance Imaging (fMRI) is a non-invasive technique used to detect changes in magnetic susceptibility, which may correlate with oxygen consumption, and therefore with neural (synaptic) activity. The measured signal is termed Blood Oxygen Level Dependent (BOLD).

KEY CONCEPT 3. Electroencephalography Electroencephalography is a non-invasive method routinely used to measure changes in voltage at the scalp, which can be originated by synchronous assemblies of neurons. EEG measures changes in voltage resulting from the flow of ions implicated in the generation of action potentials. While EEG can be measured with very high temporal resolution, it is difficult to pinpoint where in the brain the voltages originate.

KEY CONCEPT 4. Convolution and de-convolution Because of the indirect nature of fMRI measurements, precisely localized neural activity (in the time domain) is measured by fMRI as widespread and lagged. Formally, neural activity time series are said to be convoluted, i.e., blurred by a moving average weighted by a kernel (in this case, the hemodynamic response function). The (difficult) operation of inverting this process is termed de-convolution.

Leaving these assumptions aside, recent studies revealed meaningful changes in functional connectivity over time. Furthermore, the analysis of single volume co-activation patterns emerged (6, 7), thus exploiting fMRI datasets up to the limit allowed by the temporal resolution of the BOLD response. However, many issues and limitations have to be considered regarding the interpretation of these findings:

1. Are temporal changes in connectivity related to respiration or heart rate fluctuations? Since fMRI measures blood flow, physiological changes could impact directly on BOLD measurements (8).
2. Are temporal changes in connectivity related to head motion? It has been shown that movements inside the scanner can strongly influence connectivity estimates (9).
3. Do apparent temporal changes in functional connectivity appear spuriously (by chance) or by meaningful neurophysiological fluctuations? Computation of short-term correlations between random noise signals can give rise to apparently complex dynamics (10, 11).
4. Are changes in functional connectivity related to cognition or behavior or do they represent changes in vigilance and arousal? The question of how ubiquitous is sleep during the resting-state is closely related to this issue (12).

In this focused review we discuss how, when faced with such questions, the combination of fMRI with other neuroimaging methods (such as EEG and MEG) can help prove or disprove the neurophysiological relevance of dynamic changes in functional connectivity. This strategy was fundamental for validating the neurophysiological origin of spontaneous activity fluctuations in resting-state fMRI measurements (13, 14) and its application to the field of dynamic functional connectivity may prove equally important. We will first review recent demonstrations of BOLD connectivity fluctuations and their relevance for understanding changes in behavior, cognition, and vigilance states, as well as alterations caused by brain diseases, while discussing the contribution of multimodal imaging to these topics. We will then review the role of multimodal recordings in understanding the different contributions to temporal changes in functional connectivity.

## Functional Connectivity Fluctuates Over Time

Extensive reviews already cover with detail the recent developments in the field (15, 16). Here we will only summarize selected results we deem important for the main topic of this review. A recent article also reviews the neural correlates of BOLD connectivity fluctuations and is a valuable complementary reading (17).

A natural starting point is the work of Chang and Glover (18) and Sakoglu and colleagues (19). The authors of the first study performed a time–frequency coherence analysis based on the wavelet transform and demonstrated time-varying connectivity of the posterior cingulate cortex (PCC) with the rest of the Default Mode Network (DMN) (18). The use of sliding windows was also introduced to quantify correlations between BOLD signals over time. In the second study, authors also used time–frequency analyses to study dynamic functional connectivity changes during an auditory oddball task, as well as the differences between a group of schizophrenia patients and healthy controls. In (20) [and subsequently in Ref. (21)] non-overlapping windows were used to show that temporal dynamics of functional connectivity have an intrinsic burstiness, which is characteristic of critical phenomena. The use of sliding windows was applied to fMRI data from anesthetized monkeys (therefore reducing movement issues) in Hutchison et al. (22). Of particular interest was the observation of periods of high (global) correlation, reminiscent of the global avalanches of activity observed in fMRI (6), MEG (23), and LFP data (24). Dynamic functional connectivity was also established in rodents, with a temporal variation from positive to negative correlation except between homologous brain areas, which exhibit a predominantly positive correlation over time (25). In humans, functional connectivity time courses were obtained (using sliding windows) between time series extracted from all regions in the Automated anatomical labeling (AAL) template (26) and widespread correlations between direct electrophysiological recordings (EEG) and dynamic functional connectivity time series were found, as well as changes due to vigilance fluctuations (27). This paper, as well as others applying similar multimodal approaches, will be discussed with more detail in the following sections.

KEY CONCEPT 5. Sliding windows Functional connectivity can be computed over a relatively short period or window of time (in the order of seconds for fMRI time series). In a sliding window analysis, this computation is repeated many times while displacing the window forward in time (either by one or many samples), therefore estimating temporal changes in functional connectivity. The window size and the overlap between windows are free parameters to be decided.

Aside from establishing the existence of fluctuations in functional connectivity, others attempted to evaluate whether short-term patterns of connectivity can be accurately classified or clustered into a discrete set of states. Clustering via k-means revealed short-term patterns of connectivity diverging from results obtained from whole recordings (28), whereas PCA revealed stereotypical “building blocks” of short-term whole functional connectivity (29). A more straightforward clustering of dynamic functional connectivity patterns was performed in (30), in which all patterns between a set of four regions were enumerated. The limitation imposed on the number of regions allowed the explicit evaluation of information-theoretic quantities, which depend on sufficient sampling of the patterns visited over time. This method was then applied to characterize the temporal evolution of connectivity dynamics during the psychedelic state.

Most of the analyses outlined above were carried out using sliding windows, basically an extension of standard linear correlation and therefore inheriting its bivariate nature. Higher order correlations, however, are ubiquitous in brain activity (31) and a full understanding of transient connectivity states may not arise by considering pairwise interactions only. An exception is the extension of ICA to capture temporal independent modes (32). Another exception is the study of instantaneous activations and co-activations of brain activity (6). This approach is based on the identification of points of interest in resting-state data, which can be equivalently detected using blind de-convolution (33) or by identifying extreme events in the data (6). This multivariate approach reveals that co-activation patterns (i.e., all voxels containing an event or point of interest, which are concurrently observed in the same temporal volume) can reproduce all major Resting-State Networks (RSN) (34) as observed with ICA, but with only a fraction of the data (approximately 4%). Clustering of co-activation maps converged toward the same result (7). Recently, co-activation patterns of the PCC were used to assess dynamic changes during unconsciousness as induced by propofol (35).

## Behavioral and Cognitive Correlates of BOLD Connectivity Fluctuations

The human brain is inherently dynamic and variable, with oscillations over different frequencies paralleling changes in brain states (36). Non-static functional connectivity between BOLD time series is to be expected. However, it is important to understand how the intrinsic variabilities of brain activity and connectivity correlate with changes in behavior, cognitive states and environmental interactions. This is the next natural step in the investigation of dynamic functional connectivity and remains to be explored with detail.

A relevant finding is the correlation between changes in functional connectivity of the DMN and stimulus-independent thought, as demonstrated in (37). Authors performed resting-state fMRI recordings while intermittently probing mind wandering. Short-term functional connectivity of the DMN was obtained over 30 s. windows and a positive correlation between its temporal variance and an index of day-dreaming frequency was found during rest. This is an interesting example of how assessing the temporal variability of functional connectivity can assist in the interpretation of temporal variability in cognition and behavior.

In another study, spatiotemporal ICA was introduced to capture the temporal evolution of networks in task fMRI data (38). This analysis (uninformed of task time courses and the regions involved) was able to capture the transitions between task and rest, suggesting a well-defined functional role for coupling fluctuations between regions. A similar blind analysis of dynamic connectivity changes associated with task performance was performed for EEG recordings (39).

One natural question is whether spontaneous fluctuations in connectivity can bias perception and action. This question has an affirmative answer for the amplitude of BOLD signals. For instance, when showing ambiguous images to subjects (faces being one of two possible interpretations) researchers found that increased activity in the fusiform face area predicted the perception of the stimulus as a face (40). How are cognition and behavior influenced by ongoing (de)synchronization of BOLD signals? One interesting possibility is the facilitation of conscious access during periods of transient “hypersynchronization” (22) in fronto-parietal networks. It is known that conscious access elicits sustained neuronal responses propagating beyond sensory cortices and results in a massive synchronization of brain activity (41, 42). A highly connected brain state could facilitate this propagation, thus predicting conscious perception of stimuli flashed at the threshold for awareness. This directly suggests an experiment that could be carried out to probe the relationship between transient global connectivity and perception (for instance, by adapting the experimental paradigm in (40) allowing for enough time to estimate baseline functional connectivity prior to the presentation of the stimuli).

Multimodal imaging (for instance, simultaneous EEG–fMRI recordings during rest) can offer insights on the functional role of spontaneous fluctuations in functional connectivity. A rich literature demonstrates how band-specific spontaneous changes in EEG oscillatory power can bias behavior and perception. Following the previous example, pre-stimulus gamma band in the lateral part of the occipital cortex can predict conscious awareness (43). As discussed in the next section with more detail, a positive correlation between fronto-parietal BOLD connectivity and gamma power measured from occipital electrodes has been shown (27). While indirect, these are first steps in the direction of establishing a relationship between transient patterns of large-scale functional connectivity and behavior, using EEG features as a bridge between these two. We note that direct experiments should be carried out to verify such relationships, for example, by probing how baseline functional connectivity can influence conscious perception [as suggested in the previous paragraph, by adapting the paradigm in Ref. (40) and attempting to predict conscious perception from large-scale connectivity prior to the presentation of the stimuli].

## Changes in Functional Connectivity Over Time Track Brain States

In line with the possibility of connectivity indexing fluctuations of attention and perception, we recently suggested that temporal changes of functional connectivity track more radical departures from wakefulness, i.e., toward drowsiness and sleep (12).

Implicit to the use of single connectivity estimates over extended resting-state sessions is the assumption that the brain state of subjects will be homogeneous and will not depart from an idealized “resting-state wakefulness.” While the existence of the latter is disputed (as subjects will naturally exhibit different thought patterns, levels of anxiety, attention, etc.), brain states associated with considerable neurophysiological changes (such as sleep) should be monitored and avoided. This is supported by many reports showing different BOLD functional connectivity and dynamics during drowsiness and sleep (44–47).

Dynamical changes in functional connectivity can be used to decode sleep stages [as defined by AASM rules (48)]. This was first validated using EEG–fMRI recordings during sleep (49), furthermore, this classifier was then applied to fMRI resting-state studies acquired without simultaneous EEG to demonstrate the general pervasiveness of sleep during rest (12).

One lesson of these studies is that temporal fluctuations in functional connectivity can be accounted (at least partially) by changes in vigilance levels and sleep stages. Vigilance shifts indeed occur during rest, therefore it is in the interest of researchers to maintain a steady brain state during fMRI acquisition (e.g., wakefulness) in order to avoid sleep-related confounds. This is of particular importance when comparing different clinical populations (it is known that neurological and psychiatric diseases are characterized by disturbances in sleep patterns (50, 51) in addition to those caused by medication).

Arguably, loss of vigilance and sleep onset are not the only factors contributing to spontaneous fluctuations in functional connectivity. Instead, subtler changes in cognitive states can be decoded from connectivity patterns [see for instance the results in Ref. (52)]. Thus, understanding their prevalence during rest and confining analyses to particular cognitive states could reduce the possibility of false positives and negatives when comparing different populations (especially in a clinical context).

## Alterations of Dynamic Functional Connectivity in Disease

Functional connectivity reflects to a large extent the underlying structural connectivity of the brain (53). Therefore, pathological alterations in the latter could be in principle detected by static functional connectivity analyses. It is also possible that pathological alterations in functional connectivity occur on a faster time scale and thus that averaging over many minutes obscures the differences. This could be the case for diseases associated with paroxysmal events, such as temporal lobe epilepsy and absence seizures. Indeed, dynamical changes in connectivity parallel the pre-ictal and post-ictal periods during absence seizures (54) (suggesting an ictal inhibition of DMN connectivity) as well as inter-ictal spikes in temporal lobe epilepsy (55). Increased temporal fluctuations of hippocampal connectivity in temporal lobe epilepsy likely reflect spike-induced variability (56). These examples show the potential of simultaneous EEG–fMRI to integrate large-scale BOLD connectivity with faster information provided by EEG

The understanding of other diseases could benefit as well from analyses of dynamic changes in connectivity. In a recently published work, the short-term functional connectivity of schizophrenic patients (57) was studied. Clustering of connectivity patterns revealed abnormalities in some transient states but not in others. Furthermore, transition probabilities between states were altered for schizophrenic patients, suggesting a different dynamical exploration of the repertoire of possible connectivity states. Future work using combined EEG–fMRI recordings should further characterize these abnormal transient states and their link to analogous electrophysiological features [such as EEG microstates (58) and spectral changes].

## Correlations between Functional Connectivity and Band-Specific Oscillatory Power

A straightforward correlation between fMRI functional connectivity and an electrophysiological measure can be obtained from band-specific EEG power extracted from different topographical locations, which indexes the local synchronization of neurons at specific frequencies (59). This was the approach followed in Ref. (27), as well in Ref. (60) and (61). In the first study, widespread correlations between fMRI functional connectivity changes over time and EEG power were observed. These were positive for the gamma band (>40 Hz) and negative for slower frequencies including the alpha rhythm (8–12 Hz). This is in line with the hypothesized role of oscillations in these frequencies. Gamma frequency is linked to the binding of information between distant cortical areas (thus enhancing large-scale connectivity) (62). On the other hand, the alpha rhythm has an inhibitory role, suppressing those connections that are irrelevant for the current demands (63). The main limitation of this analysis is volume conduction in scalp EEG recordings, imposing limitations on the localization of EEG power fluctuations correlated with BOLD functional connectivity. Still, this is an important first step to establish the neurophysiological basis of dynamic functional connectivity. No correlations were found between dynamic functional connectivity and fluctuations in heart rate/respiration/head motion (27). With respect to changes in vigilance, subjects drifting between wakefulness and light sleep showed a different pattern of correlations between dynamic functional connectivity and EEG. A positive correlation with the slower delta band (<4 Hz) and diminished positive correlations with the gamma band were found (27).

**Figure 1:**
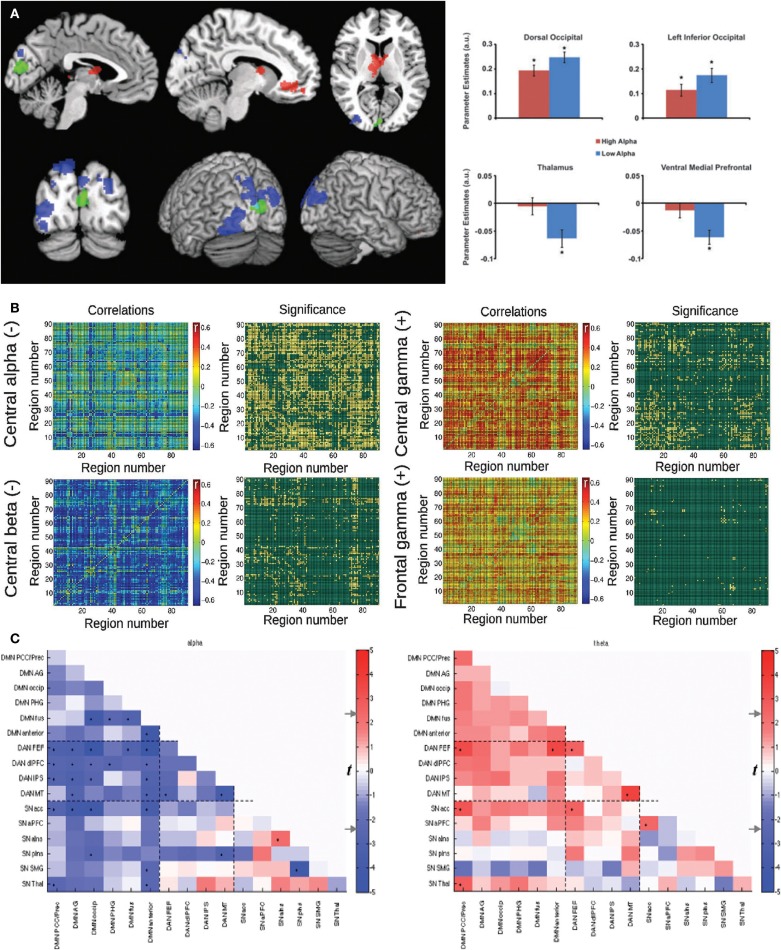
**Three studies reporting an inverse correlation between BOLD functional connectivity fluctuations and power in the alpha (8–12 Hz) band in human subjects**. **(A)** Left: brain regions showing a negative (blue) and positive (red) correlation between functional connectivity with the seed (in green) and posterior EEG alpha power. Right: Parameter estimates for high and low alpha conditions for the significant clusters depicted in the spatial map. Reproduced with permission from Ref. (60). **(B)** Mean correlation values and statistical significance of the correlation between functional connectivity (all pairs in the AAL template) and power in different EEG frequency bands: central alpha/beta (negative correlations) and central/frontal gamma (positive correlations). Reproduced with permission from Ref. (27). **(C)** Significance of correlation (in t-values) between time-varying functional connectivity and alpha (left) and theta (right) EEG power for 16 regions of interest. Reproduced with permission from Ref. (61).

Consistent results were independently reported in Ref. (60) and (61). In the first paper, psychophysiological interaction (PPI) analysis was applied to study the relationship between alpha power and connectivity of a region located in the occipital cortex. A negative correlation with alpha (but not with other bands) was found, which was also interpreted by the authors as inhibition by alpha oscillations. The second study reported a negative modulation (i.e., anticorrelation) of fMRI connectivity between the DMN and the Dorsal Attention Network (DAN) with alpha power. The correlations between fMRI dynamic connectivity and EEG power fluctuations for these studies are shown in Figure 1.

Correlations between both functional connectivity and BOLD signal amplitude (13) with EEG power in the alpha band can be reconciled given that simultaneously activated brain regions will be functionally connected when examined using very short window lengths. In other words, the shorter an observational window, the smaller will be the conceptual difference between co-activation and functional connectivity. It might be the case that correlating electrophysiological data with time-varying functional connectivity will yield a more meaningful fusion model than the direct correlation of EEG band power with the BOLD signal amplitude, the latter being a limiting case of the former.

To overcome the limitations of volume conduction, concurrent direct electrophysiological recordings and BOLD signals were obtained in the somatosensory cortex of rats (64). The results diverge from those reported in humans (27, 61) in the observation of positive correlations with all frequency bands, including the alpha and gamma bands. It must be noted that this work assessed interhemispheric correlations only. Differences could arise due to the presence of source mixing and volume conduction in EEG, absent when signals are recorded on-site via invasive electrodes. Finally, animal studies are usually performed under anesthesia whereas data from humans are commonly acquired during wakefulness, which could also explain the discrepancies. These results were partially concordant with those from another animal study (in this case in monkeys) in which a positive contribution of relatively slow (<20 Hz) LFP signals to BOLD connectivity was observed, but excluding a contribution from gamma (65). Concurrent fMRI and intra-cranial electrophysiological recordings can contribute to understanding the divergences between animal and human studies.

## Correlations between Functional Connectivity and Band-Specific Coherence

The studies mentioned in the preceding section do not directly relate measures of electrophysiological synchronization (i.e., coherence) to BOLD functional connectivity between pairs of regions. For this purpose is necessary to source localize the EEG/MEG scalp sensor data, allowing more accurate estimation of the coordinates from where time series are extracted. To study the correlation between static BOLD functional connectivity and electrophysiological coherence is not necessary to perform simultaneous combined measurements, instead, both metrics can be obtained during two different offline recordings and subsequently correlated. This approach was followed by Brookes and colleagues (66) using non-simultaneous MEG and fMRI data. Authors observed the strongest correlations when studying the beta band and when focusing on the sensorimotor cortex. A recent study overcame the limitations of offline measurements by combining simultaneous EEG–fMRI with beamforming for source localization (67). Connectivity between time series was computed via the precision matrix (i.e., the inverse of the covariance matrix) and the inference of fMRI connectivity from EEG (and vice-versa) was assessed using sparse canonical correlation analysis combined with cross-validation. The inference of fMRI connectivity from EEG connectivity was better than the inverse (EEG from fMRI) across all bands, suggesting a loss of important information in the BOLD signal for the purpose of assessing connectivity.

## Electrophysiological Correlates of fMRI Co-Activation Patterns

The patterns observed in resting-state activity reflect to a large extent those observed during task execution and sensory stimulation. For instance, by analyzing resting-state fMRI data and fMRI activation maps from a large database (*BrainMap*, www.brainmap.org) (2) were able to show a striking correspondence between both sets of independent components (RSN and the BrainMap ICA). Furthermore, recent results show that these patterns do not arise only when averaged over extended scanning sessions but voxels also become spontaneously co-activated (i.e., their activity is jointly over a certain fixed threshold) at individual volumes (6, 7). Thus, brain activity is a dynamic succession of activated/de-activated voxels and the patterns of spontaneous activations reproduce task-induced activations. This method for studying resting-state recordings resolves “instantaneous” (i.e., up to the temporal resolution of the BOLD response) and dynamic changes in co-activation.

It is yet necessary to establish the electrophysiological signatures of these spontaneous activation patterns. Besides the need to distinguish meaningful neuroelectrical activity from other confounds due to brain vasculature, head motion, etc., there is a rich history in cognitive neuroscience assigning band-specific frequencies and Evoked Reponse Potentials (ERPs) to particular tasks which, in turn, could be associated with these patterns of spontaneous co-activations. To the present day, average activity in RSN was linked to characteristic distributions of EEG frequencies (14), but temporal signatures associated with particular patterns of instantaneous co-activations remain to be studied. Furthermore, a recent study reported temporally unstable correlations between EEG signals and RSN time series, which could arise due to an intermittent manifestation of these networks (68).

The study of such correlations corresponds to an fMRI-informed EEG analysis, which is exactly the opposite of the most common strategy (i.e., using EEG as a regressor in the analysis of fMRI data). The main difficulty is the relative temporal imprecision of fMRI recordings, which complicates EEG analysis triggered by fMRI events. The improvement of fMRI sampling rates could reveal stable electrophysiological signatures of RSN in the temporal domain, by averaging temporal EEG data temporally locked to the spontaneous activation of different RSN. However, the use of scanning sequences with shorter TRs (e.g., multiband sequences) has intrinsic limitations (such as hemodynamic convolution and the possibility of heterogeneous hemodynamic coupling throughout the cortex).

## Limitations and Caveats

Two inter-related problems arise when studying temporal changes in BOLD functional connectivity. First, how to adequately define states which through their temporal progression can be used to characterize dynamics. Second, the problem of spurious results when sliding windows is used to obtain temporally evolving connectivity.

Due to poor temporal sampling it is difficult to naturally divide an fMRI resting-state session into segments of different connectivity. Interesting advances have been made by means of different analytical methods [constrained matrix factorization (69), automatic estimation of sliding window parameters using cross-validation (70), variable parameter regression and Kalman filters (71)]. Since BOLD functional connectivity is correlated with band-specific EEG power fluctuations (which admit a better temporal characterization) (27, 61), an interesting possibility is to use multimodal information to find the temporal scale at which BOLD connectivity fluctuations predominantly occur. This could be useful if, for instance, changes in connectivity were to arise mainly due to vigilance fluctuations.

Lacking a principled definition to identify temporal blocks of different connectivity, many authors have resorted to the use of sliding windows of fixed length. The second problem relates to spurious (non-biological) fluctuations in connectivity arising in this type of analyses. Recent work has shown that spurious changes in connectivity appear if the sliding window length is shorter than the largest period present in the signals (11). Window lengths at least larger than the reciprocal of the minimum frequency of interest in the signal are recommended. Considering the frequencies relevant to the resting-state BOLD signal, window lengths in the range 30–60 s are recommended for typical acquisition parameters. Another consequence of using sliding windows is low-pass filtering of connectivity time courses (with a frequency cutoff equal to the reciprocal of the sliding window length). This means that the connectivity time course (i.e., the successive correlations estimated from sliding windows as they are moved in time) will not capture high-frequency connectivity fluctuations. When correlating dynamic BOLD connectivity with signals from other modalities [such as EEG power in Ref. (27)] it is important to also perform a windowed averaging of the data, ensuring that signals will have comparable frequency content.

## Discussion and Outlook

In this review we have discussed some of the main findings on multimodal imaging of dynamic functional connectivity and their implications. A number of studies converge toward widespread correlations between fluctuations in functional connectivity and EEG or LFP band-specific power, although with different results for animal and human studies, most notably, with absent negative correlations with the alpha band in animal studies (rats and monkeys). These studies may help assuage concerns about artifacts influencing changes in connectivity over time. They suggest a dynamical nature for the coupling between regions, which is a natural constraint for interacting with an equally dynamic world. Positive correlations with the gamma band and negative correlations with the alpha band give further support to this notion. While correlations with heart rate and respiration fluctuations were not directly observed, it is known that these can nevertheless be correlated with EEG power time series (72), therefore their influence cannot be completely ruled out.

The correlation between BOLD functional connectivity and EEG synchronization measured between a pair of regions requires more sophisticated preprocessing, including source localization. Studies to date have not analyzed short-term, dynamically fluctuating connectivity but longer time series, either offline [with MEG, (66)] or with simultaneous EEG-fMRI acquisition (67). In this last study, it was observed that EEG connectivity could more accurately infer fMRI connectivity than vice-versa, raising the question of the necessity of fMRI measurements, provided accurate source localization from EEG or MEG data. Indeed, these imaging methods have many advantages over fMRI to quantify dynamic changes in connectivity, including the dissociation of band-specific contributions and the possibility of shorter temporal windows due to very high sampling rate. A main disadvantage is the relative inadequacy to resolve subcortical sources. There is also a rich literature on the large-scale organization of the human brain as measured with fMRI, since the study of large-scale patterns of brain activity (such as RSN) was pioneered using this methodology. This opens the way for the expansion of these results in a dynamical sense, but it is also important to combine these analyses with contributions from other imaging methodologies due to the reasons mentioned above. Finally, we note that even though fMRI data are poorly sampled (in the temporal sense), it is still capable to capture short-lived events as well as fast electrophysiological frequencies (such as gamma) (14).

Given the limited temporal resolution of fMRI data, the study of single volume co-activation patterns is of substantial interest. At the core of this method is the realization that patterns appearing on average are also manifest “instantaneously” as groups of jointly activated voxels. The study of these patterns holds promise to map the spontaneous cognitive processes underlying the resting-state. The combination with simultaneous EEG could reveal signatures disentangling transient artifacts from real cognitive processes. Also, observing a correlation between different conscious contents and rapidly shifting patterns of co-activated regions could strengthen the observation that functional connectivity reflects mind wandering or day-dreaming (37).

We want to note that simultaneous EEG–fMRI acquisition can pose difficult challenges [for detailed reviews see Ref. (73, 74)]. While advanced recording and preprocessing techniques can reduce EEG artifacts due to the time-varying magnetic fields of fMRI, a critical assessment of spurious residual EEG signals is always necessary. Therefore, simultaneous EEG–fMRI experiments should only be performed when absolutely necessary.

In summary, the reviewed articles show widespread evidence that: (i) functional connectivity fluctuates over time in short temporal windows, (ii) these fluctuations are neural in origin and paralleled by electrophysiological changes, and (iii) these electrophysiological correlates in humans are consistent with their purported role in binding/inhibitory processes. While some studies suggest that MEG and EEG are in general terms superior for tracking functional connectivity changes over time, the use of fMRI is still more common due to its ability to consistently map stable, high-resolution large-scale networks. Ideally, the advantages of both methods (and not their disadvantages) will be combined in the future to gain a deeper understanding of dynamic functional connectivity.

## Conflict of Interest Statement

The authors declare that the research was conducted in the absence of any commercial or financial relationships that could be construed as a potential conflict of interest.
